# Fitness After Stroke Trial (FAST): Protocol for a Preliminary Efficacy Study of Recumbent Stepper High-Intensity Interval Training

**DOI:** 10.1007/s44200-026-00104-3

**Published:** 2026-06-01

**Authors:** Bria L. Bartsch, Amanda J. Britton-Carpenter, Amanda Engler, Tyler Baldridge, Alexandra Moores, Emily M. Hazen, Robert N. Montgomery, Sandra A. Billinger

**Affiliations:** 1 Department of Neurology, University of Kansas Medical Center, 3901 Rainbow Blvd, Mail Stop 3051, Kansas City, KS 66160 USA; 2 Department of Physical Therapy, Rehabilitation Science, and Athletic Training, University of Kansas Medical Center, Kansas City, KS USA; 3 Department of Biostatistics and Data Science, University of Kansas Medical Center, Kansas City, KS USA; 4 University of Kansas Alzheimer’s Disease Research Center, Fairway, KS USA; 5 Department of Physical Medicine and Rehabilitation, University of Kansas Medical Center, Kansas City, KS USA; 6 Department of Cell Biology and Physiology, University of Kansas Medical Center, Kansas City, KS USA

**Keywords:** Stroke, Cerebrovascular accident, Vigorous intensity, Aerobic fitness, Cardiorespiratory, Arterial stiffness

## Abstract

**Background:**

Cardiorespiratory fitness and vascular health are significantly impaired post-stroke. High-intensity interval training (HIIT) has emerged as a promising strategy to improve walking in people with chronic stroke, but broadly integrating HIIT into stroke recovery remains limited. One key barrier is the reliance on maximal exercise testing to prescribe HIIT, which is often not feasible in clinical settings. To address this gap, the Fitness After Stroke Trial (FAST) evaluates the preliminary efficacy of HIIT compared to moderate-intensity continuous training (MICT), using a validated submaximal exercise testing protocol to guide individualized exercise prescription in people post-stroke.

**Methods:**

FAST will enroll 50 individuals with chronic stroke into a double-blind, two-arm, parallel-group preliminary efficacy trial. Participants will be stratified by lower extremity motor function and randomized to HIIT or MICT. Exercise sessions will occur three times per week for four weeks using a total body recumbent stepper. The primary outcome is estimated peak oxygen consumption from the Total Body Recumbent Stepper (TBRS) submaximal exercise test. Secondary outcomes include middle cerebral artery velocity at rest and during exercise, flow-mediated dilation and pulse wave velocity. Tertiary outcomes include walking speed and distance.

**Discussion:**

FAST represents one of the earliest double-blind randomized trials to directly compare HIIT and MICT on a recumbent stepper in people post-stroke, using a validated submaximal exercise testing protocol to individualize exercise intensity. This study will generate preliminary effect sizes for key physiological and functional outcomes obtained with this modality, providing the critical data needed to optimize dosing strategies and power a future definitive trial.

**Trial Registration:**

NCT05936008. Registered 7 July 2023, Study Details|Fitness After Stroke Trial|ClinicalTrials.gov.

## Background

### Background and Rationale

Cardiorespiratory fitness, measured as peak oxygen consumption (VO₂_peak_), is a critical marker of cardiovascular health with lower VO_2peak_ values associated with an increased risk of all-cause mortality [1]. People with stroke typically exhibit reduced VO₂_peak_ and often fall below their age- and sex-matched peers [2]. People with stroke often experience broader cardiovascular dysfunction, including impairments in both cerebrovascular and peripheral vascular regulation [3–5]. Our prior work has consistently demonstrated alterations, including a blunted middle cerebral artery velocity (MCAv) response to acute exercise in subacute (3 months) and chronic stroke (*≥* 6 months post-stroke) [3–8]. In addition, our systematic review and meta-analysis demonstrated that peripheral vascular function is significantly impaired post-stroke [9]. Although moderate-intensity continuous training (MICT) is recommended to improve VO_2peak_ and cardiovascular health post-stroke [10, 11], recent emerging evidence indicates high-intensity interval training (HIIT) may elicit greater physiologic benefits, particularly when exercise intensity is a key driver of fitness gains [12]. 

Despite growing support for aerobic exercise, including HIIT, to improve cardiovascular health post-stroke, implementation in clinical practice remains limited. Key barriers include reliance on maximal exercise testing and a lack of accessible tools to guide safe and individualized prescription [10, 11, 13]. To address these challenges, this trial will utilize the Total Body Recumbent Stepper (TBRS) submaximal exercise test to predict maximal power output and heart rate, allowing clinicians to prescribe precise training intensities tailored to each individual’s capacity [13]. Achieving vigorous-intensity workloads through HIIT may provide an optimal stimulus for improving both cerebro- and cardiovascular health post-stroke.

The Fitness After Stroke Trial (FAST) will evaluate and compare the effects of HIIT and MICT on cerebrovascular hemodynamics at rest and during an acute exercise bout as well as on peripheral vascular measures such as flow-mediated dilation (FMD) and pulse wave velocity (PWV) pre- and post-intervention. Together, these complementary outcomes will provide a comprehensive assessment of how different aerobic intensities influence cardiovascular function post-stroke. By pairing a clinically feasible exercise testing protocol with robust physiological outcomes, FAST is positioned to advance science and the implementation of post-stroke exercise interventions.

### Objectives

The primary objective of this study is to evaluate the preliminary efficacy of a 4-week HIIT intervention, compared to MICT, using the TBRS to improve cardiorespiratory fitness in individuals with chronic stroke. Secondary objectives are to evaluate the preliminary efficacy of HIIT on: (1) cerebrovascular hemodynamics, measured by MCAv at rest and during acute exercise; (2) Peripheral vascular function, assessed by FMD, and (3) Arterial stiffness, assessed by PWV. Tertiary objectives include examining the impact of HIIT on the 10-meter and 6-minute walk (6MWT) tests. We will explore global and regional cerebral blood flow using magnetic resonance imaging (MRI).

### Hypotheses

Post-intervention, HIIT group will demonstrate preliminary evidence of greater improvements than the MICT group in: (1) Cardiorespiratory fitness, as indicated by predicted oxygen consumption during the TBRS submaximal exercise test; (2) MCAv at rest and during acute exercise, (3) FMD, (4) PWV, (5) 10-meter walk test and 6MWT, and (6) global and regional cerebral blood flow.

## Materials and Methods

### Trial Design

This study is a two-arm, parallel-group, preliminary efficacy trial registered at ClinicalTrials.gov (NCT05936008; registered July 2023; version 3.0, April 2025). Ethical approval was granted by the University of Kansas Medical Center (KUMC) Institutional Review Board (IRB #STUDY00147598).

Given the nature of exercise interventions, complete participant blinding to perceived exertion is not feasible. Accordingly, the trial employed (1) full blinding of outcome assessors and (2) data analysts. Outcomes (Table 1) will be assessed pre- and post-intervention by assessors blinded to group allocation.

**Fig. 1:**
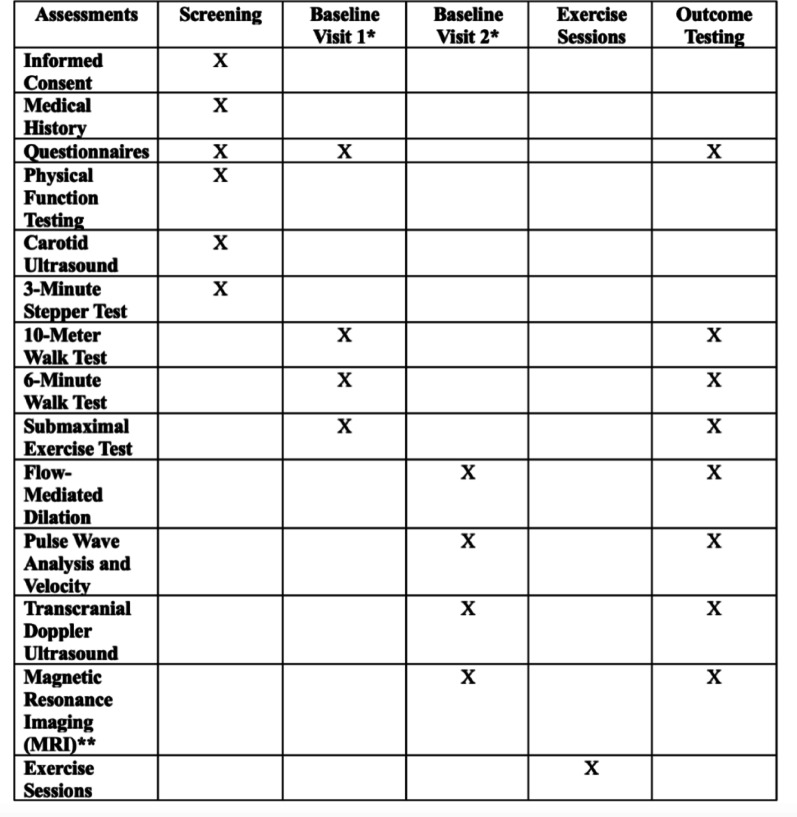
SPIRIT checklist for study procedures. *Baseline visit 1 and baseline visit 2 may be performed in opposite order if necessary for scheduling. **Magnetic resonance imaging procedure will not be completed if contraindications are present

**Fig. 2:**
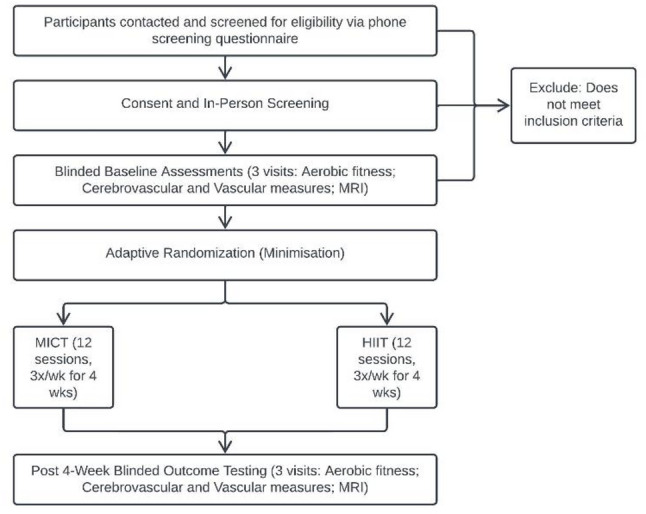
Study flow diagram. *Wk* week

During the informed consent process, participants will be informed that they will complete supervised exercise training on a seated stepper. Participants will not be informed of the intervention labels (HIIT or MICT), specific structures such as interval or continuous training, or the comparative study hypotheses, in order to minimize expectancy bias. Study procedures and flow are depicted in Figs. 1 and 2.

**Table 1:** Overview of outcomes

Variable	Details	Unit
Primary outcomes		
Predicted Peak VO_2_	Assessing change in predicted peak oxygen uptake during the TBRS submaximal exercise test	mL kg^1^ min^−1^
Secondary outcomes		
MCAv	Assessing change in MCAv response to an acute moderate-intensity exercise bout and MCAv at rest	Cm s^−1^
Flow-mediated dilation	Endothelial vascular function	%
Pulse wave velocity	Arterial stiffness	M s^−1^
Additional outcome measures		
Walking endurance	6-minute walk test	m
Gait speed	10-meter walk test	M s^−1^
Global and regional cerebral blood flow	Magnetic resonance imaging	

*Peak VO*_*2*_ Peak oxygen uptake; MCAv Middle cerebral artery velocity; *MoCA* Montreal Cognitive Assessment; *mL* milliliter; *min* minute; *kg* kilograms; *cm* centimeter; *s* second; *m* meter

### Site

Intervention and outcome assessments will be conducted at the University of Kansas Medical Center and MRI will be performed at Hoglund Biomedical Imaging Center.

### Recruitment Strategy

Recruitment will leverage existing infrastructure established through our Stroke Recovery Registry, which maintains a HIPAA-secure database of people with stroke. We contact potentially eligible individuals to gauge interest in FAST, and if the person meets basic eligibility criteria, we schedule an in-person visit for informed consent and baseline screening.

Recruitment materials such as IRB approved fliers are shared with healthcare partners across the Kansas City metro, community talks, and through digital outreach.

### Participant Eligibility

Inclusion/exclusion criteria are presented in Table 2.

**Table 2:** Inclusion and exclusion criteria

Inclusion criteria	Exclusion criteria
Both sexes between the age of 20–85 years at time of consent Chronic ischemic or hemorrhagic stroke 6 months to 15 years at time of consent. Index stroke or recurrent stroke on same side as index stroke will be allowed Ability to walk overground with assistive devices and no continuous physical assistance from another person to perform tests for gait speed and six-minute walk test Exercise continuously for minimum of 30 watts for 3 min on the recumbent stepper to demonstrate ability to perform the exercise test No aerobic exercise contraindications or other safety or physical concerns during the submaximal exercise test Able to communicate with investigators, follow 2-step command, and correctly answer consent comprehension questions Currently participating in less than 150 min of physical activity/week assessed by the Rapid Assessment of Physical Activity Stable blood pressure and statin medication doses for 30 days prior to enrollment due to effects on vascular health and hemodynamics	Hospitalization for cardiac or pulmonary disease within past 3 months Implanted pacemaker or defibrillator limiting exercise performance Reported pain that limits or interferes with activities of daily living and physical activity or exercise Severe lower extremity spasticity (Ashworth > 2) Recent history (< 3 months) of illicit drug or alcohol abuse or diagnosis of significant mental illness Major post-stroke depression (Patient Health Questionnaire, PHQ-9 ≥ 10) Currently participating in physical therapy targeting lower extremity function or another interventional study that may influence study outcomes Other significant neurologic, orthopedic, or peripheral vascular conditions that would limit exercise participation Oxygen-dependent chronic obstructive pulmonary disease Diagnosis of other neurologic disease (Multiple Sclerosis, Alzheimer’s disease, Parkinson’s disease) Self-reported pregnancy

#### Primary Outcome

The primary outcome, VO₂_peak_ post-intervention, will be predicted using the TBRS submaximal exercise test [14, 15]. Cardiorespiratory fitness is significantly reduced post-stroke with VO₂_peak_ values below the first percentile for fitness when compared to age- and sex-matched peers [2]. Lower VO₂_peak_ is associated with reduced mobility and independence, and increased mortality, underscoring the need for targeted interventions to improve cardiorespiratory fitness post-stroke [10, 16–18]. VO_2peak_ is often measured during a maximal exercise test with gas analysis and trained personnel and is often a barrier to conducting exercise tests [10, 11]. To address these challenges, our team developed the TBRS submaximal exercise test to predict VO_2peak_ [13] without requiring gas exchange analysis or maximal exertion.

Participants were informed not to consume food or drink (except water) within 2–3 h of the exercise tests and avoid caffeinated products for 6 h before the exercise test. Participants were asked to avoid vigorous physical activity for 24 h before testing. Height, weight, heart rate (HR), and blood pressure (BP) were obtained before exercise testing. Participants were fitted with a HR monitor for continuous use during the submaximal exercise test. Following our established methods [13], participants begin the exercise test at 30 watts and step between 90 and 100 spm. Workload is increased in standardized, fixed increments at 3-minute stages according to the published TBRS submaximal exercise testing protocol [13, 19–21] until the participant: (1) reaches 85% of heart rate maximum, (2) completes the test, (3) requests to stop, or (4) demonstrates absolute exercise testing termination criteria [22]. Next, we will use our online tools to predict: (1) VO_2peak_, https://biostats-shinyr.kumc.edu/Neurology_Estimate_VO2/ and (2) peak watts, https://biostats-shinyr.kumc.edu/Neurology_Estimate_Watts/ [14, 15]. For this study, we will also measure oxygen consumption using a metabolic measurement system (ParvoMedics Inc., Sandy, UT) during the TBRS submaximal exercise test to characterize physiological responses across submaximal exercise intensities and to provide a greater understanding of gas exchange during submaximal exercise pre- and post-intervention.

#### Phone Screening

A phone screen will determine age, time post-stroke, ability to walk 30 feet without assistance from another person, physical activity level, use of stable blood pressure and statin medications, hospitalization for cardiac or pulmonary disease in the past 3 months, presence of a pacemaker or defibrillator that limits exercise performance, presence of pain which interferes with activities of daily living or physical activity or exercise, current participation in physical therapy or another study which may influence study outcomes, presence of another neurologic condition other than stroke or a condition which may limit exercise participation, presence of oxygen-dependent chronic obstructive pulmonary disease, or self-report pregnancy.

### Initial Screening Visit

The initial screening visit assessments will be completed in the order below.

#### Informed Consent

Trained personnel will obtain written informed consent prior to study procedures. Consent forms will be stored in a locked cabinet.

#### Questionnaires and Physical Function Testing

We will collect demographic information, medications, and complete the American College of Sports Medicine’s cardiac risk stratification screening [22]. Height and weight will be used to calculate BMI.

The 9-item Patient Health Questionnaire (PHQ-9) [23] will provide information regarding post-stroke depression [24]. The Stroke Impact Scale [25] will characterize perceived disability and quality of life. The Modified Ashworth Scale [26] will screen for lower extremity spasticity. Individuals with scores > 2 will be excluded to ensure effective exercise participation. The Fugl-Meyer Assessment Lower Extremity (FMA-LE) subscale [27] will characterize lower limb function and inform group allocation using a minimization approach(see statistical methods section).

#### Common Carotid Ultrasonography

Participants will rest in supine for 20 min [28, 29]. Using Doppler ultrasound, we will obtain two 30-second recordings of bilateral common carotid arteries approximately 1 inch distal to the carotid bulb, using an insonation angle ≤ 60º. Intima-media thickness and blood flow velocity will be determined using semi-automated edge detection software (Carotid Analyzer and Brachial Analyzer, Medical Imaging Applications, Coralville, Iowa).

#### TBRS Familiarization

Participants will complete a 3-minute TBRS exercise bout at 30 watts, 90–100 spm. This will confirm their ability to complete the first stage of the exercise test. Participants who are unable to complete this will be excluded.

### Baseline Visit 1

Vascular health assessments will be performed in a dimly-lit, temperature-controlled room (22–24 degrees Celsius).

#### Flow-Mediated Dilation (FMD)

FMD is a valid and reliable measure of peripheral vascular health with prognostic value for cardiovascular events [30]. Evidence from our laboratory [28, 31–33] and others [34–37] have demonstrated that FMD is reduced post-stroke. We will conduct procedures between 7:00–9:00am to account for diurnal variation and follow current guidelines [30]. Participants will be asked to refrain from food or tobacco products for ≥ 6 h, alcohol or caffeine for ≥ 12 h, and vigorous activity for ≥ 24 h. Participants will also be asked to withhold blood pressure or statin medications, and staff will confirm that participants followed pre-assessment instructions. Compliance or non-compliance will be documented in REDCap.

Participants will be fitted with a 5-lead ECG for gating during FMD acquisition, then rest in supine for ~ 15 min. Next, we will conduct a 1-minute baseline recording of the right brachial artery, followed by FMD. The rapid inflation pneumatic cuff, placed 1–2 cm distal to the antecubital fossa, will be inflated to 220 mmHg for 5 min. Brachial artery vasoreactivity and blood flow velocity will be recorded for 3 min following cuff deflation. Procedures will be repeated on the left arm. We will record the following parameters at baseline to ensure scientific rigor at post-intervention: gain, depth, dynamic range, and angle of insonation (≤ 60º). Data analysis will be performed using semi-automated edge-detection software (Brachial Analyzer, Medical Imaging Applications, Coralville, Iowa), to reduce investigator bias [30, 38–40]. 

#### Pulse Wave Analysis and Pulse Wave Velocity

Arterial stiffness will be assessed using pulse wave analysis and velocity (SphygmoCor, Itasca, IL), which hold prognostic value for cardiovascular health [41–44]. Specifically, pulse wave analysis assesses the central aortic waveform [45], while carotid-femoral pulse wave velocity determines arterial stiffness [43, 44]. For pulse wave analysis, a blood pressure cuff will be placed over the brachial artery and inflate 2 times per measure to provide heart rate, blood pressure, pulse pressure, arterial pressure, and augmentation index. The average values of two measures will be reported. For pulse wave velocity, a cuff will be placed around the participant’s upper thigh, and carotid pulse will be located via palpation. Distance between the carotid pulse and suprasternal notch, suprasternal notch and thigh cuff, and femoral pulse and thigh cuff will be recorded. Two recordings will be obtained and averaged. PWV will be auto-calculated using carotid-femoral PWV (m*s^−1^) = PWV distance/pulse transit time.

#### Cerebrovascular Hemodynamics

Following the arterial stiffness assessment, we will assess cerebrovascular hemodynamics using transcranial Doppler ultrasound (TCD), a noninvasive technique that allows for measurement of MCAv at rest and during exercise.

The participant will sit on the TBRS and we will locate bilateral MCAv (2-MHz, Multigon Industries Inc, Yonkers, New York). The participant will be instrumented with a: (1) 5-lead ECG system for heart rate (Cardiocard, Nasiff Associates, Central Square, New York), (2) nasal cannula for end-tidal carbon dioxide (BCI Capnocheck Sleep 9004 Smiths Medical, Dublin, Ohio), (3) finger cuff for beat-to-beat blood pressure (Finapres, Medical Systems, Amsterdam, the Netherlands). Baseline TCD parameters, gain, depth, gate, amplitude, and probe location will be recorded to ensure the same parameters post-intervention.

Participants will perform an 8-minute rest recording, then a moderate-intensity exercise bout. Identical with our previous work [3, 4, 8, 46], a practice bout of exercise will be performed prior to the exercise recording to determine target workload. Target heart rate will be determined using [HRmax - resting HR] * target intensity + resting HR, where moderate intensity is 45–55%. The recording will begin with 2-minutes of rest, followed by 6 min of exercise, from which we will calculate kinetics (baseline, time delay, tau, and steady state) for MCAv, heart rate, mean arterial pressure, and carbon dioxide. Data will be acquired using an analog-to-digital unit (NI-USB-6212, National Instruments) and custom-written MATLAB software (R2019a or higher, The MathWorks, Inc., Natick, MA), as previously published [3, 4, 8, 46–48]. 

### Baseline Visit 2

#### Questionnaires

Fatigue and decreased reported quality of life are common post-stroke [49–51]. To assess these factors, we will use the standardized Patient Reported Outcomes Measurement Information System (PROMIS) Fatigue Scale [52] and EuroQol 5 Dimension 5 Level (EQ-5D-5 L) survey [53]. 

#### Cognition

The Montreal Cognitive Assessment (MoCA) [54] and National Institutes of Health (NIH) Toolbox [55] will be administered to assess executive function, visuospatial, naming, episodic memory, attention, language, abstraction, orientation, processing speed, and inhibition.

#### Functional Mobility

To assess gait speed and endurance, we will use the valid and reliable 10-meter walk test and 6MWT, respectively [56, 57]. For the 10MWT, participants will walk at their [1] comfortable and [2] fastest, safe walking speed over a 14 m path, where the middle 10 m are timed. We will follow American Thoracic Society guidelines for the 6MWT [58] in a 30-meter hallway. Assistive devices may be used.

#### Cardiorespiratory Fitness

The TBRS submaximal exercise test [13] will be performed to predict VO_2peak_ and watts (see “Primary Outcome” section).

### Magnetic Resonance Imaging (MRI)

MRI will be used to quantify global and regional cerebral blood flow. We will use a Siemens 3T Skyra MRI scanner with T1-weighted magnetization prepared – rapid gradient echo, fluid-attenuated inversion recovery, pulsed arterial spin labeling, T2-relaxation-under-spin-tagging, and time-of-flight 3D gradient echo sequences. These sequences capture the spatial resolution of the brain, superficial lesions, arterial blood water, cerebral blood oxygen saturation, and blood flow dynamics.

### Minimisation

Participants will be allocated to group using minimisation by FMA-LE score, where participants with an FMA-LE score ≥ 21 will be classified as “high mobility” and < 21, “low mobility.” [59] Minimisation will be performed by an unblinded study team member in an R Shiny application [60] designed by our study team (co-author, RNM).The first participant will be assigned to a group at random. Subsequently, participants will be allocated using weighted randomization where the participant being assigned has an 80% chance of being allocated to the group which promotes an equal distribution of lower extremity function between groups.

### Interventions

Exercise interventions will be performed on a TBRS 3 times per week for 4 weeks with 1:1 supervision. Heart rate will be continuously monitored using a Polar H10 (Polar Electro Oy, Kempele, Finland). Blood pressure will be assessed at midpoint of the exercise session. Both groups will perform a 5-minute warm-up (30% watts_peak_) and 5-minute cool-down (20% watts_peak_). Intervention details are below. We will assess rating of perceived exertion (RPE) using the Borg 6–20 scale [61] immediately after exercise. Following cool-down, participants will rest for an additional 5 min, during which blood pressure and heart rate will be assessed to ensure values return to near resting levels.

#### HIIT

**Fig. 3:**
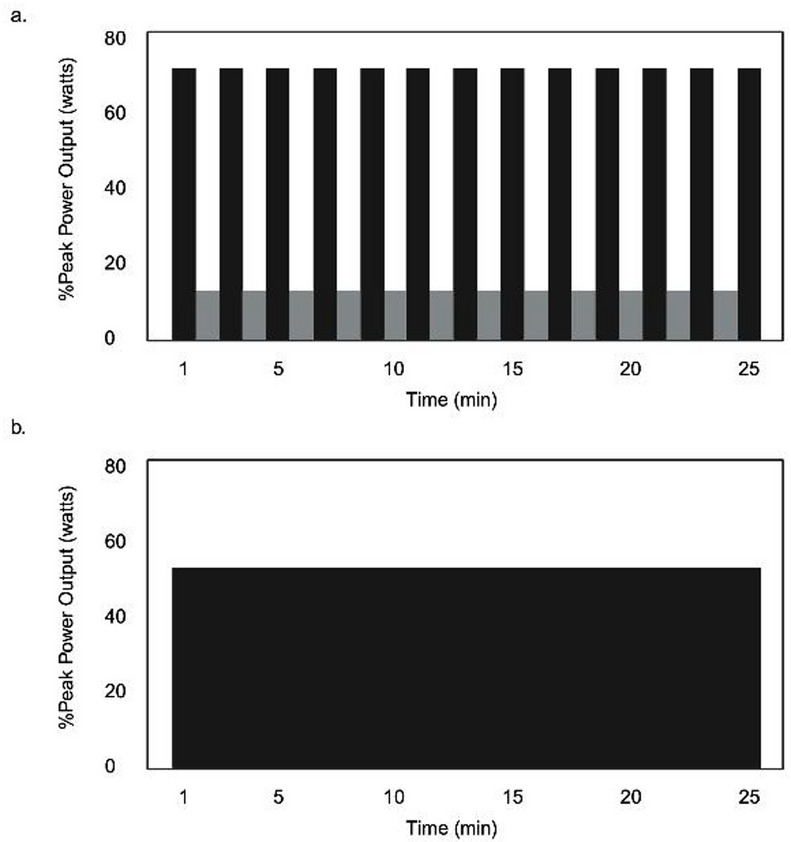
HIIT and MICT exercise protocol.** a** Conceptual depiction of exercise intensity prescription relative to peak power output measured in watts for high-intensity interval training (HIIT) and** b** moderate-intensity continuous training (MICT)

HIIT will consist of repeated 1-minute, high-intensity bouts alternated with 1-minute active recovery bouts for 25 min (Fig. 3). High-intensity will initially be prescribed at 70% watts_peak_, with a range of 65%-95% watts_peak_, to ensure that vigorous-intensity heart rates (75–85% HR_max_; 60–89% HRR) are met. The upper heart rate limit will be 85% HR_max_ in line with the TBRS submaximal exercise test. Active recovery bouts will be performed at 10% watts_peak_. The high-intensity step rate will be ~ 90–100 spm, and 50 spm for active recovery.

#### MICT

MICT will consist of continuous exercise for 25 min at 55% watts_peak_ (range: 45%-65% watts_peak_) with a target heart rate of 60–70% HR_max_ or 40–59% HRR (Fig. 3), and step rate of ~ 90–100 spm.

#### Intervention Adherence and Scientific Rigor

Exercise intensity adherence will be monitored using two methods: (1) Monitoring of heart rate during exercise and (2) Post-exercise heart rate analysis using custom R Studio [62] code. The code provides minute-by-minute minimum, maximum, and average heart rate and graphs the data with target intensity zone highlighted. This code has been developed into an open access package (https://github.com/briabartsch/ExerciseHRCode) and application (https://biostats-shinyr.kumc.edu/Neurology_ExerciseHR/).

#### Blood Lactate

Following previous procedures [48, 63], blood lactate will be measured as a surrogate marker of exercise intensity [64] at sessions #2, #5, #8, and #11 via fingerstick and lactate meter.

### Outcome Assessments

Post-intervention, the following assessments will be conducted: PROMIS Fatigue Scale, EQ-5D-5 L, MoCA, 10-meter walk test, 6MWT, TBRS submaximal exercise test, FMD, TCD, and MRI if completed at baseline. Participants will also be asked to complete the 8-item Physical Activity Enjoyment Scale (PACES-8) [65] to evaluate exercise acceptance. Every effort will be made to complete testing within 1 week post-intervention.

### Data Management

Participants will be assigned a unique identifier. Data will be stored in REDCap and on our secure network drive.

### Statistical Analysis

The primary aim of this study is to assess the preliminary efficacy of recumbent-stepper HIIT, compared to MICT, on the change in VO₂_peak_ over the 4-week intervention. We will calculate the mean, standard deviation, and 95% confidence intervals (CI) for VO₂_peak_ at each time point for groups, and the between-group difference post-intervention. We will also calculate the change from baseline to the 4-week assessment for each group. Effect size at 4 weeks will be calculated as the difference in means divided by the pooled standard deviation. Given a planned sample size of *N* = 50 with 1:1 allocation, the precision of our effect estimate will be sufficient for this preliminary efficacy trial. For example, assuming a greater improvement in HIIT (4 mL·kg⁻¹·min⁻¹) compared to MICT (2 mL·kg⁻¹·min⁻¹), and a common standard deviation of 4 mL·kg⁻¹·min⁻¹, the half-width of the 95% CI will be approximately 3.6 mL·kg⁻¹·min⁻¹.

We will fit an Analysis of Covariance (ANCOVA) model with 4-week VO₂_peak_ as the response variable. The model will be adjusted for baseline VO₂_peak_, group, and minimization variable (FMA-LE). As this study is designed to assess preliminary efficacy, we will limit adjustment and not include additional covariates. Similarly, subgroup analyses or interaction models will not be conducted due to risk of overinterpretation. Model diagnostics will be performed using residual plots, and transformations or alternative models if necessary to improve fit. The results from this trial are intended to provide directional estimates and effect size data rather than definitive efficacy, which is critical for designing a fully powered trial.

Additional outcomes, including MCAv, FMD, PWV, 10MWT, 6MWT, and MRI will be assessed in a similar way. Point estimates and 95% CIs will be calculated for the data and model-based estimates will be calculated using an ANCOVA adjusted for the same covariates as the primary outcome. Given the preliminary nature of the study, analyses will be conducted using available data without imputation, with transparent reporting of missingness and attrition.

### Safety and Adverse Event Monitoring

Adverse events will be assessed for study-relatedness, graded for severity using the National Cancer Institute Common Terminology Criteria for Adverse Events v5.0, and reported to the Institutional Review Board in accordance with University policies. Quarterly adverse event reports will be reviewed by an independent safety officer.

### Dissemination Plans

Personnel who have actively participated in study design and data acquisition will be invited to co-author findings in manuscripts, presentations, and conference proceedings.

## Discussion

The Fitness After Stroke Trial will address a critical gap in stroke recovery research by implementing a scalable, physiologically grounded protocol to examine the preliminary efficacy of HIIT and MICT on predicted VO_2peak_ in individuals with chronic stroke. This trial will be among the first to integrate cerebrovascular and peripheral vascular outcomes alongside a submaximal exercise testing approach, offering a novel and clinically feasible method for exercise prescription.

### Expanding Clinical Usability of Submaximal Exercise Testing

While maximal exercise testing is considered best practice, its feasibility in stroke rehabilitation is limited by cost, complexity, and access to equipment. Surveys of physical therapists reveal minimal use of exercise testing and concern regarding patient safety [66, 67]. However, prior stroke recovery research has confirmed the reliability and validity of submaximal exercise testing. In a study by Eng et al. [68] submaximal treadmill and cycle ergometer tests demonstrated good to excellent test–retest reliability, and VO₂ values showed strong concurrent validity with VO₂ maximum (*r* = 0.66–0.80; *p* < 0.05). The TBRS submaximal exercise test offers an additional validated assessment that eliminates the need for ECG and gas exchange analysis, expanding testing accessibility while enabling accurate VO_2peak_ prediction and individualized exercise prescription. Prescribing exercise intensity based on a percentage of maximal power output may allow for better individualization by accounting for motor impairments that influence movement efficiency [69]. As with most exercise testing and training protocols, participation in FAST requires individuals to demonstrate a minimal level of functional capacity to safely complete the initial stage of the TBRS submaximal exercise test. Accordingly, the protocol is intended for individuals with chronic stroke who can perform seated stepping exercise and may not be generalizable to those with the most severe motor impairments or limited exercise tolerance. This design choice reflects safety considerations and the study’s focus on evaluating preliminary efficacy of exercise intensity prescriptions in a population capable of engaging in structured aerobic training.

### Developing Strategies for Professionals Implementing Exercise Post-stroke

Despite increasing evidence for the benefits of aerobic exercise post-stroke, implementation remains limited due to lack of training, resources, and standardized tools available to clinicians [66, 67]. Recognizing these challenges, we developed resources to support clinical translation of the exercise strategies used in FAST. These include web-based calculators to estimate peak power output and VO_2peak_ and an open-access package [62] to assess intensity attainment. As such, the exercise program used in this trial has direct implications for clinical utilization.

Importantly, while the TBRS will be used in FAST, we recognize that not all settings may have TBRS equipment. However, because the TBRS submaximal exercise test and our exercise protocols are grounded in physiological measures such as heart rate and power output, the intervention can be adapted for use with other modalities such as a cycle ergometer.

### Considerations for HIIT Implementation Post-stroke

Moderate-intensity exercise is currently recommended for stroke recovery [10, 11], with 50% and 42.4% of inpatient and outpatient therapists reporting prescribing moderate-intensity exercise, whereas only 2.2% of inpatient and 1.7% of outpatient therapists report using high-intensity exercise [67]. However, since this work was published in 2017, there has been an increase in HIIT studies post-stroke. Despite this, few studies have implemented HIIT using the TBRS. For example, a systematic review and meta-analysis [70] of 17 studies examining the effects of exercise on brain-derived neurotrophic factor post-stroke reported one used a TBRS. Most studies continue to rely on treadmills, highlighting a persistent gap in the literature regarding accessible, safe, and scalable HIIT delivery options post-stroke.

Our laboratory previously demonstrated that an acute bout of low-volume, short-interval TBRS HIIT, prescribed using a percentage of peak power output, is feasible post-stroke [9]. Participants achieved vigorous-intensity, completed the session, and no study-related serious adverse events occurred. Building on this work, FAST represents a critical next step in evaluating whether a structured TBRS-based HIIT intervention can improve fitness and vascular function.

### Cerebrovascular Function Post-stroke

Resting MCAv is related to stroke recovery, with evidence suggesting that a lower MCAv is associated with poor functional recovery [71, 72]. More recently, acute physiologic challenges such as exercise and sit-to-stand maneuvers have been used to characterize cerebrovascular responses post-stroke, offering insight into cerebrovascular regulation. For example, evidence from our laboratory demonstrated that MCAv response to a bout of moderate-intensity exercise is blunted post-stroke when compared to age- and sex-matched controls [4, 8, 29]. This attenuation was further illustrated in a longitudinal case study of an individual with two sequential strokes [4]. Following the initial stroke, the affected hemisphere showed a ~ 50% reduction in MCAv amplitude. After the second stroke, an 81% reduction in MCAv amplitude was observed in the newly affected hemisphere, with further decline on the original side. These findings emphasize the profound impact of stroke on cerebrovascular hemodynamics and importance of evaluating whether interventions, such as HIIT, can enhance cerebrovascular function.

### Peripheral Vascular Function Post-stroke

Research from our laboratory [28, 31–33, 73] and others [34–37] have demonstrated that peripheral vascular function is impaired post-stroke. This dysfunction may hinder recovery and increase the risk of recurrent cardiovascular events [30, 71, 72, 74, 75]. Notably, impaired FMD in the acute phase of stroke independently predicts new-onset cardiovascular events [74] and is associated with greater disability 3 months post-stroke [75]. In a meta-analysis of 26 studies post-stroke, our laboratory reported an average FMD of 3.9%, indicating reduced endothelial function [33]. For comparison, a meta-analysis of over 1,500 healthy individuals and those with coronary artery disease identified an FMD threshold of 6.5%, below which vascular dysfunction is likely [76]. Importantly, aerobic exercise improves vascular function. Our previous work showed an 8-week TBRS aerobic exercise program significantly improved FMD and reduced systolic blood pressure post-stroke [13], demonstrating that peripheral vascular function is modifiable. FAST builds upon this work by testing whether different exercise intensities elicit distinct vascular benefits post-stroke.

### Trial Status

Study recruitment began June 2023 and study completion was December 2025. Protocol version: 3.0, April 2025.

## Data Availability

Once the trial is complete, the minimum data set will be made available on Open Science Framework.

## Abbreviations

ECG: Electrocardiography
EQ-5D-5L: EuroQol 5 dimension 5 level
FAST: Fitness after stroke trial
FMA-LE: Fugl-Meyer assessment lower extremity subscale
FMD: Flow-mediated dilation
HIIT: High-intensity interval training
HRR: Heart rate reserve
HR_max_: Maximal heart rate
MCAv: Middle cerebral artery blood velocity
MICT: Moderate-intensity continuous training
MoCA: Montreal cognitive assessment
MRI: Magnetic resonance imaging
PACES-8: 8-item physical activity enjoyment scale
PHQ-9: 9-item patient health questionnaire
PROMIS: Patient reported outcomes measurement information system
PWV: Pulse wave velocity
TBRS: Total body recumbent stepper
TCD: Transcranial doppler ultrasound
VO_2_: Oxygen consumption
6MWT: 6-minute walk test
10MWT: 10-meter walk test
